# Prevalence and Knowledge of Hepatitis B Virus Infection among Pregnant Women in the Ningo-Prampram District, Ghana

**DOI:** 10.1155/2020/7965146

**Published:** 2020-04-27

**Authors:** Precious Kwablah Kwadzokpui, Elliot Elikplim Akorsu, Albert Abaka-Yawson, Solomon Sosu Quarshie, Stephen Adomako Amankwah, Philip Apraku Tawiah

**Affiliations:** ^1^Department of Medical Laboratory Sciences, School of Allied Health Sciences, University of Health and Allied Sciences, Ho, Ghana; ^2^Department of Pharmacognosy and Herbal Medicine, School of Pharmacy, University of Health and Allied Sciences, Ho, Ghana

## Abstract

**Background:**

Hepatitis B virus (HBV) infection has been suggested to play a role in various adverse birth outcomes. The study determined the prevalence as well as knowledge of hepatitis B virus infection among pregnant women in the Ningo-Prampram District of the Greater Accra Region of Ghana.

**Materials and Methods:**

A cross-sectional study using simple random sampling technique was used to recruit 213 pregnant women receiving antenatal care in three different health facilities (Prampram Polyclinic (PPC), Dangme Community Hospital (DCH), and Old Ningo Health Center (ONHC)) in the Ningo-Prampram District of Ghana from November 2018 to January 2019. A semi-structured questionnaire was used to collect data which included participants' HBsAg test results, sociodemographic and gynaecological characteristics, and their level of knowledge on HBV infection. Knowledge of the participants on HBV infection was classified as either excellent, good, or poor based on their cumulative percentage scores from the questionnaire according to Al Rubaish system of classification.

**Results:**

Overall low-intermediate prevalence of HBV infection was 3.3%; however, PPC recorded the highest prevalence of 4.0% while DCH and ONHC recorded 2.82% and 2.50%, respectively. Statistically significant association was observed between HBV infection and the health facility. Majority (77.40%) of the study participants had poor knowledge on HBV infection while only 14 (6.57%) had excellent knowledge on HBV. Regarding excellent knowledge, 8 (11.0%) among the participants were demonstrated by the majority of those who received antenatal care from DCH. Generally, knowledge on HBV and the infection was poor among the study participants. Knowledge on HBV infection was found to be associated with residential status (*p* = 0.006), educational level (*p* < 0.001), occupation (*p* < 0.001), and gestational period (*p* < 0.001). Participant's knowledge was also significantly associated with the health facility (*p* = 0.027).

**Conclusion:**

HBV infection among pregnant women is prevalent in the Ningo-Prampram District even though the prevalence is not very high. The majority of pregnant women in the Ningo-Prampram District inadequate knowledge on HBV infection and it mode of transmission. Intensive public health education on the HBV infection is required in the district to help prevent and manage future transmissions as well as inform the population about the negative side effects of the virus and the need to prevent it by way of vaccination.

## 1. Background

Infection with hepatitis B virus (HBV) results in significant human morbidity and mortality, primarily through the aftermath of chronic infection [1]. Chronic hepatitis B infection which embraces a large spectrum of the disease remains a serious public health problem globally with about 350-400 million people affected [2] and causing deaths ranging from 600,000 to 650,000 annually [3–5]. Studies have indicated that HBV infection remains a global challenge, with one-third of the world's population having serological evidence of current or previous infection [6] and progression of the disease towards cirrhosis, liver failure, or hepatocellular carcinoma occurring in 15-40% of infected subjects [7]. The prevalence of HBV infection is a significant public health concern [8] especially in pregnant women who are HBV carriers since vertical transmission from an infected mother to her unborn child remains the predominant mode of transmission from an infected mother to the unborn child [9]. Nearly 400 million people are chronically infected with hepatitis B virus (HBV) worldwide [6, 10–12], and almost half have acquired their infections either through mother to infant transmission or in early childhood, especially in countries where HBV has intermediate to high prevalence [6, 13]. In a study conducted in Obudu, Southern Nigeria, out of 836 pregnant women who were screened for HBsAg, 55 representing 6.6% tested positive for the virus with the mean age and parity of the seropositive subjects being 26.9 ± 5.0 (range between 18 and 38 years) and 2.2 ± 1.2 (range between 0 and 5), respectively [14]. The trend of infection in comparison to the prevalence of 6.6% as determined by Utoo in similar studies within Nigeria over the years seems to be increasing with Port Harcourt recording a prevalence of 4.3% [15], Enugu 4.6% [16], and Ibadan in southwestern Nigeria 8.3% [17]. In Ghana, some studies on the prevalence of HBV infection among pregnant women have been carried out in different parts of the country with varying prevalence rates reported. These include a prevalence of 10.6% recorded in the Eastern Region of Ghana [18], 9.5% in the Asante Akim North District of the Ashanti region [19], 12.6% in the Department of Obstetrics and Gynecology, Komfo Anokye Teaching Hospital, Kumasi, Ghana [20], 2.4% in Ho Municipal Hospital Antenatal Clinic in the Volta region of Ghana [21], and 16.7% in Eikwe, Western Region, Ghana [22]. In a survey to assess the knowledge of attitudes and practices of hepatitis B management of pregnant women and healthcare practitioners at Komfo Anokye Teaching Hospital in Kumasi, Ghana, over 20% (44 of 209) of the women were uncertain that HBV could be transmitted to their children, almost 23% (47 of 209) of them had no knowledge of how HBV could be transmitted, and more than 50% were unaware that HBV could be transmitted from a mother to her unborn child [23]. Similar studies conducted in the Kintampo North District of the Brong Ahafo Region of Ghana to assess the level of knowledge of pregnant women about HBV infection showed that the level of awareness was low among pregnant women with over 50% of the respondents reporting not to be aware of HBV [24]. In people with lowered immunity such as pregnant women, cases of HBV infection need to be well established and made known to the target population in order to avoid the detrimental outcomes it poses on the affected individual. Studies on the case of HBV infection and knowledge about the disease among pregnant women in the Ningo-Prampram District are, however, non-existent.

## 2. Materials and Methods

### 2.1. Study Design/Eligibility Criteria

A cross-sectional study using simple random sampling technique was employed to ascertain the prevalence and knowledge of HBV infections among pregnant women in the Ningo-Prampram District of the Greater Accra Region of Ghana from November 2018 to January 2019. The study was carried out at the antenatal clinic of three major health facilities within the Ningo-Prampram District, i.e., Prampram Polyclinic Clinic (PPC), Dangme Community Hospital (DCH), and Old Ningo Health Center (ONHC) around the coastal belt of the district generally within a separation range of about 4.2 km to 11.5 km apart. The district housing of these hospitals lies on latitude 5°42′54^″^*N* and longitude 0°6′22^″^E, i.e., 5°42′54^″^N 0°6′22^″^E. Pregnant women aged ≥15 years, either pregnant for the first time and/or had been pregnant before and were registered attendees at the antenatal clinics of the three selected health facilities, were recruited in the study. Pregnant women who had been vaccinated against HBV infections as well as pregnant women who registered with the clinics but were not residents of the district were excluded from the study.

### 2.2. Questionnaire Administration

A semi-structured questionnaire designed to capture participant's HBsAg test results, demographic data, and questions on HBV and its infection, was administered by the researcher to collect the data for this study. Socio-demographic details obtained for this study include the age, residential and educational status, occupation, marital status, and religious affiliation as well as parity and gestational period of the participants. Questions on HBV infections, transmission, and prevention were posed to the participants by the researcher in the language (Dangme, Twi, Hausa or English) understood by the participant to ascertain their knowledge about the disease. Knowledge of the participants on HBV infection was classified as either excellent, good, or poor based on their cumulative percentage scores from the questionnaire according to Al Rubaish system of classification where a score of >80% means excellent knowledge, 60-80% means good knowledge, and <60 means poor knowledge [25].

### 2.3. Laboratory Analysis

About 2-3 ml of venous blood sample was obtained by a qualified laboratory scientist at the laboratory units of the three health facilities into appropriately coded plain tubes (red top sample tubes contain no anticoagulant) employing the standard blood collection procedures and then allowed to clot. The samples were then separated by centrifugation to obtain the serum for testing for the presence of HBsAg. Upon successful separation of the serum, 2-3 drops of the serum were transferred onto the sample pad of the Micropoint HBsAg gold rapid screen test kit (Trinity Biotech Plc, Japan) using the manufacturer's attached pipette. Observation and reading of the result were done within 5-20 minutes according to the manufacturer's instructions. For purposes of certainty in result outcome, test strips were quality controlled using known HBsAg-positive serum samples stored by the facility laboratories for the same purpose on clinical samples. In addition, all HBsAg-positive results were repeated to rule out possible test kit fault. Negative result was indicated by the appearance of a pink band in the control (C) region while a positive result was indicated by the appearance of a second band in addition to the band in the control region at the test (T) region. The results of the tests were recorded in the antenatal folders of the study participants and captured onto the questionnaire.

### 2.4. Data Handling and Management

Unique codes were assigned to each participant taking into consideration the location of the health facility to ensure participants' data security and confidentiality. Additionally, information recorded in the questionnaire was checked for completeness and entered using fit for purpose designed excel forms into Microsoft Office Excel 2016 and data generated (qualitative data) were analyzed using Statistical Package for the Social Sciences (SPSS version 22.0). Descriptive methods of analysis such as tables and graphs were generated to illustrate the occurrence of the various factors in the study. Categorical data was analyzed using the chi-square test (*χ*^2^) and Fisher's exact *t*-test from GraphPad Prism version 6.00 for windows (GraphPad software, San Diego USA). A *p* value of <0.05 was considered statistically significant.

### 2.5. Ethical Consideration

Ethical clearance was obtained from the Research Ethics Committee (REC) of the University of Health and Allied Sciences (UHAS) with Ethical Clearance Certificate Number UHAS-REC A.4 [162] 18-19. Approval was also obtained from the Ningo-Prampram District Health Directorate and the Administration of the three hospitals [Prampram Polyclinic (PPC), Dangme Community Hospital (DCH), and Old Ningo Health Center (ONHC)]. In addition, written consent was obtained from all participants who agreed to partake in the study after they were thoroughly informed and sensitized about the study.

## 3. Results

### 3.1. Demographic Characteristics of Respondents

A total of 213 pregnant women were enrolled in the study. Their ages ranged from 15 to 41 years with the median age being 27 years. One hundred and ninety-five (89.20%) of the total participants lived in rural areas of the district. Majority of the participants (105, 49.30%) only had basic education, worked in the informal sector (60.10%), and were married (57.70%) as at the time of this study. Generally, the participants were Christian religion dominated (198, 93.00%) and were multiparous (had birthed about 2 to 4 children). At the time of the study, 92 (43.2%) of the study participants were in their second trimester while 88 (41.3%) and 33 (15.5%) were in their third trimester and first trimester, respectively.

### 3.2. Prevalence of HBV Infection

**Figure 1:**
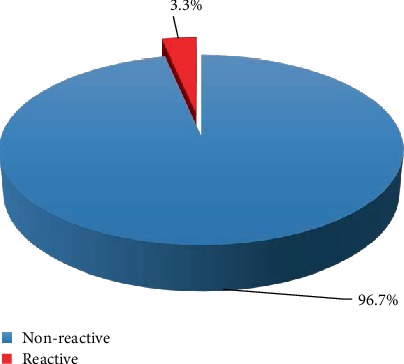
HBsAg status indicating an HBV infection prevalence of 3.3%.

Out of the 213 pregnant women whose HBsAg status were obtained, 7 (3.30%) were reactive (positive) for the virus while 206 (96.70%) were nonreactive (negative) indicating a prevalence of 3.3% of HBV infection among pregnant women who received antenatal care at the selected Antenatal Clinics in the Ningo-Prampram District of the Greater Accra Region of Ghana (Figure 1).

### 3.3. Prevalence of HBsAg among Pregnant Women Based on Health Facility

**Figure 2:**
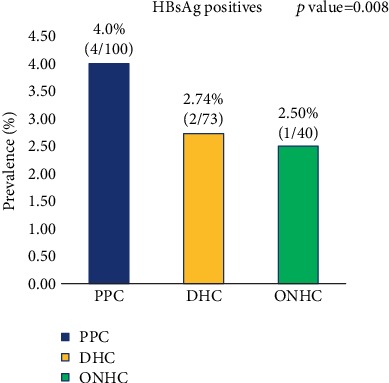
Prevalence of HBsAg among pregnant women stratified by health facility.

Based on each health facility, Prampram Polyclinic recorded the highest prevalence of 4.0% while Dangme Community Hospital and Old Ningo Health Clinic recorded 2.74% and 2.50% HBV prevalence, respectively. Prevalence category recorded in all three facilities was not different from the overall low-intermediate prevalence observed generally. Statistically significant difference was observed between HBV prevalence and the health facilities (Figure 2).

### 3.4. Association between Sociodemographic Factors and Seroprevalence of HBsAg

In this study, out of the total number of participants aged 30-39 years, five (9.09%) tested positive for HBsAg whereas two (25.00%) of the study participants aged forty years and above tested positive for HBsAg. HBsAg prevalence was highest among participants from the urban area (4.35%) compared to those who lived in the rural community (3.16%). Pregnant women who had their secondary education were recorded to have higher prevalence (5.66%) compared to those who had their basic education 3 (2.86%); however, the illiterates had the second highest prevalence (3.13%). Participants who were in their third trimester had a prevalence of 4.55% higher than their counterparts in their second trimester while those who had given birth to more than five children (grand multiparous) presented with the highest prevalence of 10.00%. Among the sociodemographic and obstetrical characteristics of pregnant women assessed in this study, only age (*p* < 0.01) was significantly associated with HBsAg infection. Compared to participants aged <20 years, those forty years and above were 11.92 times more likely to be infected with HBV (OR = 11.92, 95% CI = 0.5–284.30) whereas pregnant women who belonged to the traditional religion were 5.12 times more likely to be infected by HBV than their Muslim and Christian counterparts (OR = 5.11, 95% CI = 0.23-115.98). In addition, gravid women who are grand multiparous were 19.59 times likely (OR = 19.59, 95% CI = 0.90–425.94) to be infected by HBV compared to the primiparous participants. However, all risk assessments computed were not statistically significant (Table 1).

### 3.5. Percent Response of Participants on HBV and the Infection

Of the 213 study participants, 42 (19.72%) correctly indicated that hepatitis B was a virus while only 37 (17.37%) rightly affirmed that HBV affects the liver but 69.01% did not know that HBV could affect just anyone. A hundred and sixty-two (76.06%) out of the 213 participants did not know that an infected person is capable of living without ever presenting with complaints related to the infection whereas a smaller 4.23% incorrectly claimed that hepatitis B could not affect the health of the unborn child as well as their own health. Forty-two (19.72%) of the 213 study participants affirmed that HBV could cause liver cancer while 26.29% knew that HBV could be prevented by vaccination. However, 16 (7.51%) thought that eating well could prevent one from HBV infection (Table 2).

### 3.6. Knowledge on HBV Infection Stratified by Sociodemographic Characteristics

Pregnant women's knowledge on HBV infection was classified as either excellent, good, or poor based on their cumulative percentage scores from the questionnaire (>80% = excellent, 60 − 80% = good, and <60 = poor) [25]. Out of the 213 study participants, 14 (6.57%) were excellent in knowledge about HBV infection, and 34 (15.96%) had good knowledge about HBV infection whereas the majority 165 (77.46%) were poorly informed on HBV infections. Factors that significantly influenced a participant's knowledge on HBV infection were residential (*p* = 0.006) and educational (*p* < 0.001) statuses and occupation (*p* < 0.001) and gestational periods (*p* < 0.001). Findings of this study revealed that majority of pregnant women who demonstrated excellent knowledge lived in the rural areas (17.39%), had their tertiary education (9, 39.13%), worked in the formal sector (31.82%), and were in their first trimester (33, 52.38%) (Table 3).

### 3.7. Participant's Knowledge Stratified by Health Facility

**Figure 3:**
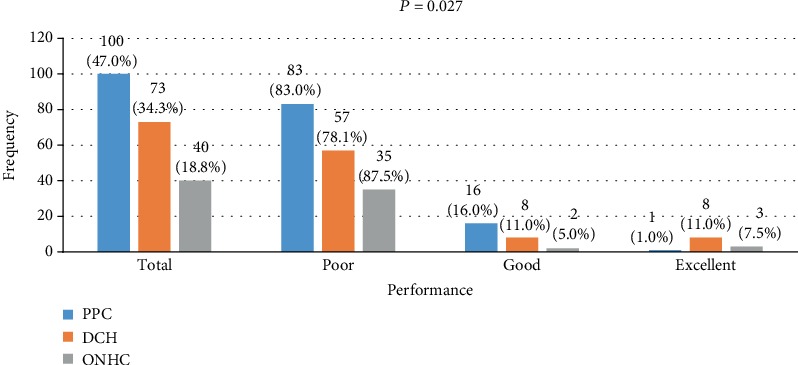
Participants' performances stratified by health facility.

Out of the 213 participants recruited for this study, 47% were surveyed from PPC while 34.3% and 18.8% were surveyed from DCH and ONHC, respectively. In the majority of the participants who demonstrated excellent knowledge about HBV infections, 8 (11.0%) were pregnant women who received antenatal care from the DCH. Eleven percent and 16.0% of the participants who received antenatal care from DHC and PPC, respectively, had a good knowledge about the infection. In general, knowledge was largely poor across all the three health facilities. Statistically significant association exists between participant's performance on knowledge about HBV infection and the health facility (Figure 3).

### 3.8. Association between Participants' Knowledge and HBsAg Prevalence among Pregnant Women

Out of the 213 pregnant women interviewed, none of the overall 6.57% participants who demonstrated excellent knowledge concerning HBV infection was seropositive for HBsAg. Five (14.71%) out of the 34 (15.96%) who demonstrated good knowledge had the highest percentage of HBV infection while 2 (1.21%) of the 165 (77.46) who demonstrated poor knowledge had the least prevalence. There was a statistically significant association between the knowledge of the study participants and HBV infection indicating that one's knowledge about the disease influences the persons' chance of exposure or infection (*p* ≤ 0.001) (Table 4).

## 4. Discussion

This study found an overall low-intermediate (2%-4.99%) [26] HBV prevalence of 3.3% among pregnant women in the Ningo-Prampram District. Though Ghana among other African countries is considered an HBV endemic area [27, 28], the current study suggests that individual districts may have different prevalence rates. Comparable intermediate prevalence rates of 2.4% and 2.2% among pregnant women attending antenatal clinic in the Volta Region and Yilo Krobo District, Eastern Region of Ghana, respectively, have been documented [18, 21]. However, studies in other parts of Ghana recorded higher prevalence rates such as 16.7% in Kumasi [23], 10.6% in the Eastern Region [18], and 9.5% in the Asante Akim North Municipality of the Ashanti region [19]. Similarly, Central Nigeria reported a prevalence of 19.5% [29] while Cameroon recorded 7.85% [30].

It is worth noting that there exists a worldwide age-associated decline in the prevalence of hepatitis B virus infection [31], which may be ascribed to the institution of HBV testing in pregnant women combined with immunoglobulin prophylaxis and/or hepatitis B vaccination immediately after delivery in children born to HBsAg-positive mothers [21, 30]. This law, passed in 1991, mandated the universal vaccination of infants and 12-year-old adolescents on a national scale and a mandatory screening for HBsAg of pregnant women in order to identify babies in need of treatment with hepatitis B immune globulins (HBIG) and vaccine at birth [32]. This therefore indicates that indeed by the time this study was being carried out, majority of the gravid women in this study (median age = 27 years, minimum age = 15 years, and maximum age = 41 years) might have benefited from this program hence contributing to the low-intermediate prevalence documented in the findings of this study.

Similarly, the Global Advisory Group of the Expanded program on Immunization recommended the integration of hepatitis B vaccine into national immunization systems in all countries with a hepatitis B carrier prevalence (HBsAg) of 8% or greater by 1995 and in all countries by 1997 [32, 33] which hence could also be a contributing factor for the low-intermediate prevalence recorded since these guidelines have been duly inculcated into the health policy management by Ghana Health Service.

Among the three health facilities used in this study, PPC recorded the highest prevalence rate of 4.0% while DCH and ONHC recorded comparable prevalence rates of 2.7% and 2.5%. Additionally, the relationship between HBV infection and the hospital facility was statistically significant (*p* = 0.008). The observed differences in prevalence may be due to differences in the quality and implementation of health education and policies in the individual health facilities.

Among the sociodemographic and obstetrical characteristics of pregnant women assessed in this study, only age (*p* < 0.01) was significantly associated with HBsAg infection, a finding that contradicts a study by Younmo and colleagues among similar populations in the Eastern Region of Ghana who found no influence of age on HBV infection [18]. However, similar studies conducted in the Volta region revealed a similar finding as observed in this study where age was a significant contributor of HBV infection among pregnant women. In fact, same studies reported that chances of women between 46 and 50 years of age becoming HBsAg carriers were high [21], further supporting the findings in this study where though not statistically significant, the computed odds ratio revealed that pregnant women aged forty years and above were 11.92 times more likely at risk of HBV infection (OR = 11.92, 95% CI = 0.5–284.30).

Key attribute required in eliminating the transmission cycle of the HBV in newborns is the expectant mothers' knowledge on the viral infection. Unfortunately, majority of the participants in this study had only their formal education up to the basic level, some of which probably could not even complete. As a result, their knowledge on HBV was generally poor. About half of the study participants indicated no knowledge of hepatitis B virus infection prior to the interview section which was revealed in wrong responses to the basic questions presented them. Similar observation was recorded at Mbagathi District Hospital in Nairobi, Kenya, where 144 (50.2%) had no knowledge of HBV infection before the study [34]. This difference in knowledge and precisely the low level of awareness of HBV are due to the lack of formal education about HBV as compared to other diseases of similar modes of transmission and burden. Comparable to other studies, majority of the respondents did not know that most HBV carriers are asymptomatic and could transmit the virus to uninfected persons through sexual intercourse [35]. In fact, personal observations during the study period indicated largely that some of the study participants might have been exposed greatly to HBV infection as a result of some relatives losing their lives due to chronic HBV infection.

Discouraging outcome on a participant's level of knowledge on risk factors of HBV infection was realized. The woman's inability to identify the various risk factors that predisposes her and her developing foetus to HBV infection further makes her more vulnerable to acquiring the infection and accounts largely for the increased risk of exposure that is being reported by many research works in similar population across the globe [16–19]. This shortfall could be as a result of their low level of formal education which according to the findings from this study has a significant influence (*p* < 0.001) on their overall knowledge on HBV infection. Lack of consistent and accurate public sensitization by health officials on the infection can also be blamed for this outcome. The low level of knowledge among pregnant women presents a possible threat due to the potential spreading of the HBV to sexual partners, newborns, the community, and the country at large. This is most especially true if the woman is also a carrier of the HBeAg owing to the fact that infants born to HBeAg-positive mothers are likely to be infected and with a 95% chance of progressing to chronicity [36].

This study recorded a significant relationship between participant's performance and health facility attended (*p* = 0.027). Majority of the pregnant women who received antenatal care from DCH demonstrated excellent knowledge compared to those recruited from PPC and ONHC. Generally, participants who received antenatal care from PPC were the poorest in terms of knowledge, followed by that of DCH and ONHC. This outcome could be due to a situation of the busier the hospital, the less time made by the health professionals of the hospital to engage the patients in health education during their regular antenatal visits.

In this study, no HBsAg positivity was recorded for participants who demonstrated excellent knowledge on HBV and the infection which is indicative of the fact that adequate knowledge on the disease is prerequisite to combat the infection among pregnant women and the general population. Participant's knowledge was significantly associated with HBV infection among pregnant women in the Ningo-Prampram District.

## 5. Conclusion

HBV infection among pregnant women is prevalent in the Ningo-Prampram District even though the prevalence is not very high. Majority of pregnant women in the Ningo-Prampram District do not know much about HBV infection and how it is transmitted. Intensive public health education on the HBV infection is required in the district to help prevent and manage future transmissions as well as inform the population about the negative side effects of the virus and the need to prevent it by way of vaccination.

## 6. Limitation

This study was not able to use a more reliable testing techniques such as ELISA and/or PCR but employed the use of commercial HBsAg test kits instead in testing for the presence of HBsAg among the study participants. The study also did not conduct an HBV profile test to determine HBeAg status of participants in order to ascertain their ability to transmit the infection to others.

## Figures and Tables

**Table 1:** Association between sociodemographic factors and seroprevalence of HBsAg.

Parameters	HBsAg positive	HBsAg negative	OR (95% CI)	*p* value
	7 (3.29)	206 (96.71)		
Age				
<20	^†^	15 (100.00)	1	<0.001
20-29	^†^	135 (100.00)	0.11 (0.00–5.97)	
35-39	5 (9.09)	50 (90.90)	3.38 (0.18–64.54)	
≥40	2 (25.00)	6 (75.00)	11.92 (0.5–284.30)	
Residence				
Rural	6 (3.16)	184 (96.84)	1.79 (0.21–15.22)	0.751
Urban	1 (4.35)	22 (95.65)	1	
Educational status				
None	1 (3.13)	31 (96.88)	2.24 (0.08–57.43)	0.618
Basic	3 (2.86)	102 (97.14)	1.60 (0.08–32.14)	
Secondary	3 (5.66)	50 (94.34)	3.26 (0.16–65.64)	
Tertiary	^†^	23 (100.00)	1	
Occupation				
None	^†^	63 (100.00)	0.35 (0.01–18.39)	0.090
Formal	^†^	22 (100.00)	1	
Informal	7 (5.47)	121 (94.53)	2.78 (0.15–50.38)	
Marital status				
Single	^†^	26 (100.00)	1	0.071
Married	7 (5.69)	116 (94.31)	3.41 (0.19–61.62)	
Cohabiting	^†^	64 (100.00)	0.41 (0.01–21.25)	
Religion				
Christian	7 (3.54)	191 (96.46)	1	0.760
Muslim	^†^	13 (100.00)	0.95 (0.05–17.46)	
Traditional	^†^	2 (100.00)	5.11 (0.23–115.98)	
Parity				
Primiparous	^†^	72 (100.00)	1	0.062
Multiparous	5 (4.13)	116 (95.87)	6.85 (0.37–125.65)	
Grand multiparous	2 (10.00)	18 (90.00)	19.59 (0.90–425.94)	
Gestational period				
1st trimester	^†^	33 (100.00)	1	0.458
2nd trimester	3 (3.26)	89 (96.74)	2.62 (0.13–52.09)	
3rd trimester	4 (4.55)	84 (95.45)	3.57 (0.19–68.11)	

Data is presented as figure and percentage in parentheses. ^†^No positive case recorded. *p* value significant at <0.05.

**Table 2:** Percent response of participants on HBV and the Infection.

Questions	Responses		
	Yes	No	Do not know
Is hepatitis B a virus?	42 (19.72)	14 (6.57)	157 (73.71)
Does hepatitis B affect the liver?	37 (17.37)	19 (8.92)	157 (73.71)
Can hepatitis B be transmitted through unsterilized needles, blades, and other sharp material?	53 (24.88)	11 (5.16)	149 (69.95)
Can contaminated blood and blood products transmit hepatitis B?	59 (27.70)	7 (3.29)	147 (69.01)
Is hepatitis B transmitted through unsafe sex?	53 (24.88)	10 (4.69)	150 (70.42)
Can hepatitis B be transmitted through kissing?	33 (15.49)	22 (10.33)	158 (74.18)
Can an infected person remain without symptoms?	45 (21.13)	15 (7.04)	153 (71.83)
Can hepatitis B affect any person?	59 (27.70)	7 (3.29)	147 (69.01)
Will an infected person remain infected for life?	26 (12.21)	25 (11.74)	162 (76.06)
Can you be infected with HBV by shaking hands with infected person?	19 (8.92)	42 (19.72)	152 (71.36)
Is a specific diet required for all infected persons?	28 (13.15)	25 (11.74)	160 (75.12)
Can hepatitis B infection cause liver cancer?	42 (19.72)	6 (2.82)	165 (77.46)
Can hepatitis B affect my pregnancy and me?	56 (26.29)	9 (4.23)	148 (69.48)
Can you identify an infected person just by looking at the person?	10 (4.69)	56 (26.29)	147 (69.01)
Can hepatitis B be prevented by vaccination?	56 (26.29)	4 (1.88)	153 (71.83)
What do you do to be protected from hepatitis B virus infection?	Get vaccinated	Eat well	Do not know
	52 (24.41)	16 (7.51)	145 (68.08)

Data is presented as figure and percentages in parentheses.

**Table 3:** Knowledge of HBV infection among pregnant women by sociodemographics.

Parameter	Knowledge			
	Poor (%)	Good (%)	Excellent (%)	*p* value
	165 (77.46)	34 (15.96)	14 (6.57)	
Age				
<20	12 (80.00)	3 (20.00)	0 (0.00)	0.471
20-29	103 (76.30)	21 (15.56)	11 (8.15)	
30-39	42 (76.36)	10 (18.18)	3 (5.45)	
≥40	8 (100.00)	0 (0.00)	0 (0.00)	
Residence				
Rural	153 (80.53)	27 (14.21)	10 (5.26)	0.006
Urban	12 (52.17)	7 (30.43)	4 (17.39)	
Educational status				
None	32 (100.00)	0 (0.00)	0 (0.00)	<0.001
Basic	92 (87.62)	12 (11.43)	1 (0.95)	
Secondary	34 (64.15)	15 (28.30)	4 (7.55)	
Tertiary	7 (30.43)	7 (30.43)	9 (39.13)	
Occupation				
Unemployed	8 (44.44)	7 (38.89)	3 (16.67)	<0.001
Formal sector	8 (36.36)	7 (31.82)	7 (31.82)	
Informal sector	104 (81.25)	20 (15.63)	4 (3.13)	
Marital status				
Single	18 (69.23)	6 (23.08)	2 (7.69)	0.540
Married	93 (75.61)	21 (17.07)	9 (7.32)	
Cohabiting	54 (84.38)	7 (10.94)	3 (4.69)	
Religion				
Christian	153 (77.27)	32 (16.16)	13 (6.57)	0.999
Muslim	10 (76.92)	2 (15.38)	1 (7.69)	
Traditional	2 (100.00)	0 (0.00)	0 (0.00)	
Parity				
Primiparous	49 (68.06)	17 (23.61)	6 (8.33)	0.134
Multiparous	99 (81.82)	14 (11.57)	8 (6.61)	
Grand multiparous	17 (85.00)	3 (15.00)	0 (0.00)	
Gestational period				
1st trimester	22 (34.92)	8 (12.70)	33 (52.38)	<0.001
2nd trimester	75 (81.52)	11 (11.96)	6 (6.52)	
3rd trimester	68 (77.27)	15 (17.05)	5 (5.68)	

Data presented as frequency and percentages in parentheses. *p* value is significant at <0.05.

**Table 4:** Knowledge versus seroprevalence of HBsAg among pregnant women.

Knowledge	Total	HBsAg positive	HBsAg negative	Chi-square	*p* value
Excellent	14 (6.57)	0 (0.00)	14 (100.00)	16.659	<0.001
Good	34 (15.96)	5 (14.71)	29 (85.29)		
Poor	165 (77.46)	2 (1.21)	163 (98.79)		

Data is presented as figure and percentage in parentheses. *p* value significant at <0.05.

## Data Availability

Research data available on request.

## References

[1] MacLachlan J. H., Cowie B. C. (2015). Hepatitis B Virus Epidemiology. *Cold Spring Harbor Perspectives in Medicine*.

[2] Polish Group of Experts for HBV, Flisiak R., Halota W. (2017). Recommendations for the treatment of hepatitis B in 2017. *Clinical and experimental hepatology*.

[3] Kilonzo S. B., Gunda D. W., Mpondo B. C. T., Bakshi F. A., Jaka H. (2018). Hepatitis B virus infection in Tanzania: current status and challenges. *Journal of Tropical Medicine*.

[4] Tao I., Compaoré T. R., Diarra B. (2014). Seroepidemiology of hepatitis B and C viruses in the general population of Burkina Faso. *Hepatitis Research and Treatment*.

[5] Ma L., Norton M. G., Mahmood I. (2014). Transplacental transfer of hepatitis B neutralizing antibody during pregnancy in an animal model: implications for newborn and maternal health. *Hepatitis Research and Treatment*.

[6] Alssamei F. A. A., Al-Sonboli N. A., Alkumaim F. A. (2017). Assessment of immunization to hepatitis B vaccine among children under five years in rural areas of Taiz, Yemen. *Hepatitis Research and Treatment*.

[7] Borgia G., Carleo M. A., Gaeta G. B., Gentile I. (2012). Hepatitis B in pregnancy. *World Journal of Gastroenterology: WJG*.

[8] Ibrahim N., Idris A. (2014). Hepatitis B awareness among medical students and their vaccination status at Syrian Private University. *Hepatitis Research and Treatment*.

[9] Nguyen G., Garcia R. T., Nguyen N., Trinh H., Keeffe E. B., Nguyen M. H. (2009). Clinical course of hepatitis B virus infection during pregnancy. *Alimentary Pharmacology & Therapeutics*.

[10] Rajbhandari R., Chung R. T. (2016). Treatment of hepatitis B: a concise review. *Clinical and Translational Gastroenterology*.

[11] Said A., Jou J. H. (2014). Hepatitis B vaccination and screening awareness in primary care practitioners. *Hepatitis Research and Treatment*.

[12] Kirbak A. L. S., Ng’ang’a Z., Omolo J., Idris H., Usman A., Mbabazi W. B. (2017). Sero-prevalence for hepatitis B virus among pregnant women attending antenatal clinic in Juba Teaching Hospital, Republic of South Sudan. *Pan African Medical Journal*.

[13] Kumar M., Singh T., Sinha S. (2012). Chronic Hepatitis B Virus infection and pregnancy. *Journal of Clinical and Experimental Hepatology*.

[14] Utoo B. T. (2013). Hepatitis B surface antigenemia (HBsAg) among pregnant women in southern Nigeria. *African Health Sciences*.

[15] Akani C. I., Ojule A. C., Opurum H. C., Ejilemele A. A. (2005). Sero-prevalence of hepatitis B surface antigen (HBsAg) in pregnant women in Port Harcourt, Nigeria. *The Nigerian Postgraduate Medical Journal*.

[16] Obi S. N., Onah H. E., Ezugwu F. O. (2006). Risk factors for hepatitis B infection during pregnancy in a Nigerian obstetric population. *Journal of Obstetrics and Gynaecology*.

[17] Anaedobe C. G., Fowotade A., Omoruyi C. E., Bakare R. A. (2015). Prevalence, socio-demographic features and risk factors of hepatitis B virus infection among pregnant women in Southwestern Nigeria. *The Pan African Medical Journal*.

[18] Cho Y., Bonsu G., Akoto-Ampaw A. (2012). The prevalence and risk factors for hepatitis B surface Ag positivity in pregnant women in eastern region of Ghana. *Gut and Liver*.

[19] Ephraim R., Donko I., Sakyi S. A., Ampong J., Agbodjakey H. (2015). Seroprevalence and risk factors of hepatitis B and hepatitis C infections among pregnant women in the Asante Akim North Municipality of the Ashanti region, Ghana; a cross sectional study. *African Health Sciences*.

[20] Adjei C. A., Asamoah R., Atibila F., Ti-enkawol G. N., Ansah-Nyarko M. (2016). Mother-to-child transmission of hepatitis B: extent of knowledge of physicians and midwives in Eastern region of Ghana. *BMC Public Health*.

[21] Luuse A., Dassah S., Lokpo S. (2017). Sero-prevalence of hepatitis B surface antigen amongst pregnant women attending an antenatal clinic, Volta Region, Ghana. *Journal of Public Health in Africa*.

[22] Völker F., Cooper P., Bader O. (2017). Prevalence of pregnancy-relevant infections in a rural setting of Ghana. *BMC Pregnancy and Childbirth*.

[23] Cheng A., Jose J., Larsen-Reindorf R. (2015). A survey study of pregnant women and health care practitioners assessing the knowledge of, attitudes and practices of hepatitis B management at a teaching hospital in Kumasi, Ghana, West Africa. *Open Forum Infectious Diseases*.

[24] Abdulai M. A., Baiden F., Adjei G., Owusu-Agyei S. (2016). Low level of Hepatitis B knowledge and awareness among pregnant women in the Kintampo North Municipality: implications for effective disease control. *Ghana Medical Journal*.

[25] Al Rubaish A. (2011). The usefulness of global student rating items under end program evaluation surveys in quality improvements: an institutional experience in higher education, Saudi Arabia. *iBusiness*.

[26] Nelson N. P., Easterbrook P. J., McMahon B. J. (2016). Epidemiology of hepatitis B virus infection and impact of vaccination on disease. *Clinics in Liver Disease*.

[27] CDC (2008). *Geographic distribution of chronic hepatitis B virus*.

[28] Alberta Government (2018). *Hepatitis B Virus Infection–High Endemic Geographic Areas*.

[29] Asaga M. P., Chipago S. A., Ehi A. P. (2019). High prevalence of hepatitis B virus infection among pregnant women attending antenatal care in Central Nigeria. *Journal of Infectious Diseases and Epidemiology*.

[30] Kfutwah A. K. W., Tejiokem M. C., Njouom R. (2012). A low proportion of HBeAg among HBsAg-positive pregnant women with known HIV status could suggest low perinatal transmission of HBV in Cameroon. *Virology Journal*.

[31] Ott J. J., Stevens G. A., Groeger J., Wiersma S. T. (2012). Global epidemiology of hepatitis B virus infection: new estimates of age-specific HBsAg seroprevalence and endemicity. *Vaccine*.

[32] Romano' L., Paladini S., Zanetti A. R. (2012). Twenty years of universal vaccination against hepatitis B in Italy: achievements and challenges. *Journal of Public Health Research*.

[33] Namgyal P. (2003). Impact of hepatitis B immunization, Europe and worldwide. *Journal of Hepatology*.

[34] Ngaira J. A. M., Kimotho J., Mirigi I. (2016). Prevalence, awareness and risk factors associated with Hepatitis B infection among pregnant women attending the antenatal clinic at Mbagathi District Hospital in Nairobi, Kenya. *Pan African Medical Journal*.

[35] Frambo A. A., Atashili J., Fon P., Ndumbe P. (2014). Prevalence of HBsAg and knowledge about hepatitis B in pregnancy in the Buea Health District, Cameroon: a cross-sectional study. *BMC Research Notes*.

[36] Salman K., Rashmi S. P., Molly M., Kumar V. S., Zeenat S. (2015). Hepatitis B virus infection in pregnant women and transmission to newborns. *Asian Pacific Journal of Tropical Disease*.

